# Pathogen-Associated Molecular Patterns Induced Crosstalk between Dendritic Cells, T Helper Cells, and Natural Killer Helper Cells Can Improve Dendritic Cell Vaccination

**DOI:** 10.1155/2016/5740373

**Published:** 2016-02-11

**Authors:** Tammy Oth, Joris Vanderlocht, Catharina H. M. J. Van Elssen, Gerard M. J. Bos, Wilfred T. V. Germeraad

**Affiliations:** ^1^Department of Internal Medicine, Division of Hematology, Maastricht University Medical Centre+, P.O. Box 616, 6200 MD Maastricht, Netherlands; ^2^Tissue Typing Laboratory, Department of Transplantation Immunology, Maastricht University Medical Centre+, P.O. Box 616, 6200 MD Maastricht, Netherlands

## Abstract

A coordinated cellular interplay is of crucial importance in both host defense against pathogens and malignantly transformed cells. The various interactions of Dendritic Cells (DC), Natural Killer (NK) cells, and T helper (Th) cells can be influenced by a variety of pathogen-associated molecular patterns (PAMPs) and will lead to enhanced CD8^+^ effector T cell responses. Specific Pattern Recognition Receptor (PRR) triggering during maturation enables DC to enhance Th1 as well as NK helper cell responses. This effect is correlated with the amount of IL-12p70 released by DC. Activated NK cells are able to amplify the proinflammatory cytokine profile of DC via the release of IFN-*γ*. The knowledge on how PAMP recognition can modulate the DC is of importance for the design and definition of appropriate therapeutic cancer vaccines. In this review we will discuss the potential role of specific PAMP-matured DC in optimizing therapeutic DC-based vaccines, as some of these DC are efficiently activating Th1, NK cells, and cytotoxic T cells. Moreover, to optimize these vaccines, also the inhibitory effects of tumor-derived suppressive factors, for example, on the NK-DC crosstalk, should be taken into account. Finally, the suppressive role of the tumor microenvironment in vaccination efficacy and some proposals to overcome this by using combination therapies will be described.

## 1. Introduction

Immunotherapy aims to stimulate or modulate immune response to specifically recognize and attack transformed cells in cancer and infectious diseases. The development of cancer immunotherapy includes various strategies like recombinant protein technologies and cell-based therapies. Clinically applied cellular therapeutic vaccines are currently under development and optimization. The advantage of specific active immunotherapy using Dendritic Cells (DC) is mainly the stimulation of* de novo* antitumor immune responses and the induction of immunological memory to prevent tumor relapse. This requires the coordinated induction of innate and adaptive immune responses including Natural Killer (NK) cells, T helper 1 (Th1), and Cytotoxic T Lymphocytes (CTL). Even though the feasibility of this approach was demonstrated in several clinical studies in cancer patients, there is still need to increase its efficacy. Identifying in general how DC perceive danger signals leading to the generation of* de novo* immune responses against disease-associated antigens and which signals induce and enhance the interaction of DC with different immune effector cells is important to increase the efficacy of cancer vaccination strategies. In this paper, we will therefore briefly discuss the selection of appropriate adjuvants by reviewing the roles of PAMPs and PRRs in vaccination strategies against infectious diseases and focus on the translation of these ideas in the application of cancer vaccines.

## 2. Adjuvants: Critical Components in Vaccination

Preventing infectious diseases by means of vaccination is considered one of the biggest milestones of modern medicine, saving countless lives. Key components of vaccines are adjuvants, which are added to induce, shape, enhance, accelerate, and prolong the immune responses against a desired antigen (Ag). These immunomodulators can be divided into three classes: nonimmunogenic systems increasing the delivery of Ag to target cells and influencing Ag presentation, immunostimulatory compounds (e.g., ligands of immune receptors), and the combination of both. DC represent a crucial target of most vaccine adjuvants, in both preventive and therapeutic vaccination strategies [1, 2]. Depending on the environmental stimuli DC encounter, they transmit signals to immune effector cells inducing immunogenic or tolerogenic immune responses. Defining optimal adjuvants will lead to (a) reduction of number of immunizations, (b) ensuring a rapid response towards pathogens, (c) reduction of the amount of Ag needed, (d) broadening the induced antibody (Ab) response, and (e) directing and localizing the induced immune responses and ensuring the most effective and suitable response towards a particular Ag [3, 4].

## 3. PRR-Triggering Agents: What DC Vaccination Can Learn from Prophylactic Vaccines

Over the last decades it has become clear that adjuvants such as oil in water emulsions and alum are required for the effectiveness of vaccines against certain pathogens. However, these most frequently used adjuvants only induce suboptimal cellular immune responses. More recently, the use of selected innate triggers (pathogen-associated molecular patterns or PAMPs), which have been naturally part of live attenuated or inactivated vaccines, has been tested in clinical trials exploring the safety and effectiveness of these innate adjuvants on the promise they induce superior cellular immune responses. This concept can directly be translated to the development of DC vaccines for cancer. Such vaccines are usually generated by differentiating monocytes into immature DC [5], followed by tumor antigen loading and maturation of DC. Many different cocktails of growth factors, cytokines, and PAMPs have been used in the preparation [6–11] indicating the most optimal mixture may not have been identified yet.

One crucial step for vaccination efficacy is the induction of appropriate Th cell subsets from naïve CD4^+^ T cells. CD4^+^ T cells are important for helping cellular and humoral arms of the immune response. They are necessary for the induction of CD8^+^ T cell and B cell memory [12]. Th cell polarization is influenced by antigen presenting cells (APC), like DC. Both the subset of DC being activated and the encountered trigger will influence the fate of Th cells. Even though many promising adjuvants have been revealed in experimental studies, clinical trials with beneficial outcome are scarce (reviewed in [13]). This discrepancy is at least in part explained by crucial differences between the animal models used and the complexity of the human immune system* in vivo*. For instance, Toll Like Receptors (TLR) expression pattern and ligand specificity differ between mice and men [3, 14, 15] and within subsets of DC in men [6]. Therefore, it is important to study the polarization kinetics of naive CD4^+^ T cells by differently matured DC in an autologous human system* in vitro* [16].

Another very important parameter to consider during the selection process of an appropriate adjuvant is the promotion of NK-DC crosstalk [17]. NK-DC crosstalk amplifies Th1 responses by providing an early source of IFN-*γ* [7, 18, 19]. Vaccine injection induces upregulation of TLR on NK cells, increases activation, and enhances IFN-*γ* levels [20]. NK cells play a crucial role as amplifiers of DC-induced responses. If potent cellular responses are desired, the choice of adjuvant should have direct NK cell activating properties as well as the indirect capacity via maturation of DC and NK-DC crosstalk.

The key to determine the optimal use of TLR triggers lies most probably* in vivo*. During a pathogenic insult, the invader is able to trigger several PRRs (on various cell types) leading to the induction of multiple signaling pathways and an optimal cooperation between different immune cells. As such, several experimental studies revealed additive or synergistic activation of DC and a resulting enhanced interaction with immune cells when multiple PRR pathways were stimulated [21–25]. Therefore, there is rational to investigate which PRR triggers can be combined to activate synergistic signaling pathways. It could be plausible that several combinations will also be of inhibitory nature, an aspect that has to be prevented. Moreover, recent evidence suggests that targeting nonimmune cells, like stromal cells, influences Th1 and CD8^+^ T cell responses [26]. This complex expression pattern of TLR/PRR on various immune and nonimmune cells offers new combination strategies to maximize adjuvant capacities. As such, modern adjuvant selection could benefit from identifying potential synergic combinations of PRR triggers to enhance the induced immune responses against a particular Ag.

## 4. PAMPs in DC Vaccination to Improve Anticancer Helper T and NK Cell Responses

Since the first clinical trial with DC vaccination in 1996 [27], much effort on improving the efficacy of this potentially powerful anticancer therapeutic approach has been made. In 2010, the FDA approved the first DC-based vaccine against advanced prostate cancer [28]. This vaccine is prolonging the patient's progression-free survival for several months. Different ongoing clinical trials testing DC-based vaccination clearly exemplify the importance of DC-based vaccines in future standard treatments. Even though a stabilization of disease and prolonged survival was observed in several cases, limited effect on bulky tumors was observed [28–31]. The overall benefit on clinical outcome is around 15–20%, indicating that there is need for further optimizations.

**Figure 1:**
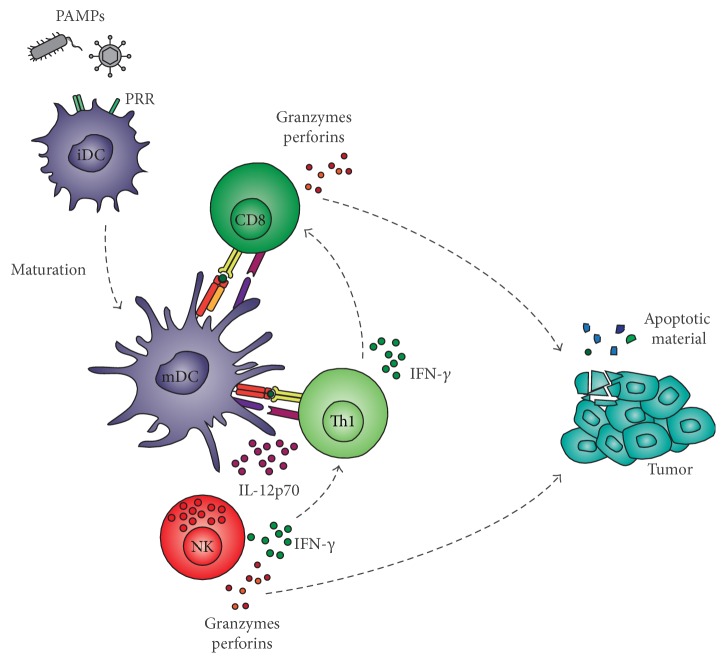
Desired immune interactions upon PAMP stimulation of immature dendritic cells. PAMPs trigger immature MoDC (iDC) to mature DC (mDC) and activate cells involved in antitumor responses (NK cells and CD4^+^ and CD8^+^ T cells). The (crucial) cytokine milieu (only IL-12 and IFN-*γ* are shown) generated by their activation can break the tolerizing effects of the TME resulting in killing of tumor cells by CD8^+^ T cells and NK cells. Apoptotic material from tumors may be taken up by resident DC and may enhance the immune response.

**Figure 2:**
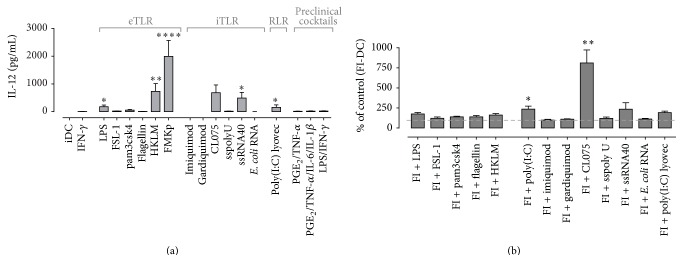
DC-derived IL-12p70 production upon single and multiple PRR triggering. (a) iDC were matured in the presence of IFN-*γ* and different PRR triggers as indicated on *x*-axis. Cytokine production was determined in the culture supernatant after 48 h of maturation. Data are represented as mean + SEM and representative of at least 6 independent experiments. Kruskal-Wallis test significance as compared to DC matured with IFN-*γ*. ^*∗*^ *P* ≤ 0.05, ^*∗∗*^ *P* ≤ 0.01, and ^*∗∗∗∗*^ *P* ≤ 0.0001. (b) Synergy of FMKp/IFN-*γ* (FI) maturation with poly(I:C) and CL075 as measured by their IL-12p70 production.

Whereas initial research focused on generating mainly tumor-specific CTL responses, it is becoming increasingly clear that the activation of multiple immune effector cells is the key to success for curative cancer vaccination.* Ex vivo*-matured DC should have the capacity to interact with endogenous immune cells of the patient to induce a potent type-1 immune response enabling the elimination of all tumor cells [32]. The criteria which a potent vaccine should fulfill are challenging and include APC activation, coactivation of CD4^+^ and CD8^+^ T cells, priming naive cells and modulating anergic memory CD8^+^ T cells, crosstalk with DC subsets and NK/NKT cells, and induction of long-lived memory. One way by which DC control and modulate adaptive immune responses is their secretion of cytokines and chemokines. Different from signal 1 (TCR-MHC) and 2 (costimulation) required for proper tumor-antigen-specific T cell activation [33, 34], signal 3 (cytokines) is not only able to polarize T helper cells into a specific lineage but can also recruit and activate other immune cells like NK cells [7, 35–40]. Furthermore, the delivery of signal 4 (homing properties) is important to ensure recruitment of activated T cells [41]. As such, the selection of appropriate PAMPs for priming of DC having capacities to induce type-1 immune responses is desired (Figure 1). We found that combinations of* Klebsiella pneumoniae* membrane fragments (FMKp, confirmed to contain at least TLR2 and TLR4 ligands [42]) and CL075 (TLR7/8 ligand) or poly(I:C) (TLR3 ligand) in combination with IFN-*γ* are the most powerful combination leading to strong Th and NK helper cell responses [8, 17, 43] (Figure 2), where IL-12 production can be used as a very important read-out marker. In the end, PAMPs can be used as modulator for* ex vivo* DC generation or in combination strategies with radiotherapy, chemotherapy, or targeted therapies in the treatment of cancer [44].

## 5. Importance of IL-12: Is It All We Need?

IL-12 is long known to be an essential factor driving Th1 responses [34, 45–47] and NK cells responses [34]. We have observed a significant positive correlation between the amount of IL-12 produced by moDC and the resulting polarization of naive CD4^+^ T cell into Th1 cells and the induction of IFN-*γ*-producing NK cells [17]. This quantitative requirement needs to be taken into account while screening for new superior maturation cocktails or methods. These findings are strengthened by recent clinical studies indicating a positive correlation between high IL-12p70-producing DC and time to progression [48, 49]. Moreover, older studies tested the systemic application of IL-12 and revealed a positive anticancer effect. However, the implementation of rhIL-12p70 in cancer treatment approaches was hindered by its dose-limiting toxicities [34, 50–55]. Altogether, these findings emphasize the use of IL-12p70-producing DC to ensure local production and delivery of this cytokine to come closer to successful vaccination strategies.

The failure of DC to produce (“high enough”) IL-12p70 could be one of the factors explaining the limited effects of DC-based vaccination clinical trials (listed in [56]). We tested various DC maturation cocktails used in clinical trials (PGE_2_/TNF-*α* [±IL-6, IL-1*β*], alpha-DC, and LPS/IFN-*γ*) and all these DC produced significantly lower levels of IL-12p70 compared to FMKp/IFN-*γ*-matured DC (Figure 2(a)). FMKp is a membrane fraction of the* Klebsiella pneumoniae* bacterium and contains at least TLR2/4 ligands [12, 30]. FMKp/IFN-*γ* DC were by far the highest IL-12 producers compared with DC stimulated with other PRR triggers in combination with IFN-*γ*. Moreover, the importance of IL-12 on* de novo* generation of Th1 responses is underpinned, as only FMKp/IFN-*γ* DC were able to polarize and induce IFN-*γ* production in naive CD4^+^ cells after coculture [16]. Such FMKp/IFN-*γ*-matured DC also induced the highest NK cell-derived IFN-*γ* production, followed by LPS/IFN-*γ* and alpha-DC-activated NK cells. Of note, soluble factors derived from PGE_2_/TNF-*α* (±IL-6 and IL-1*β*) matured DC did not lead to NK helper activation (unpublished data and [7]).

Besides the finding that PGE_2_ exerts a direct inhibiting effect on DC-derived IL-12p70 production [57], another possible explanation for the PGE_2_ cocktail not to induce IL-12 producing DC is the absence of IFN-*γ* in the maturation cocktail. It has been shown that IFN-*γ* boosts DC cytokine production [58] and additionally prevents DC from exhaustion. We demonstrated that rhIFN-*γ* dose-dependently determined the magnitude of IL-12p70 production (and production of T cell recruiting CXCL9 and CXCL10) by DC, whereas TNF-*α* had no effect on the DC-derived cytokine and chemokine production during the priming phase [17]. However, TNF-*α* was shown to be important for the upregulation of costimulatory markers on DC [59].

Different strategies to maximize the IL-12 production can be applied. One approach is the genetic manipulation of the DC* ex vivo* which was demonstrated to shape key immunological outcome parameters [60]. Another approach is the use of PRR triggers during the* ex vivo* maturation of DC. Several murine and human* in vitro* studies illustrate that the combination of multiple PRR triggers, thus engaging multiple PRR signaling pathways, leads to synergistic effects on DC maturation [8, 11, 61].

We previously showed that the strength of PRR signaling by a single trigger can considerably enhance the IL-12p70 production [16]. Furthermore, cooperative PRR signaling by using the bacterial trigger FMKp with the viral trigger poly(I:C) [17] or CL075 [8] leads to synergistic IL-12p70 production (Figure 2(b)), followed by increased helper cell induction, whereas other combinations did not. This approach requires a thorough search for the most optimal combination of PAMPs of different origins (bacterial, viral, and fungal) or triggering different PRR families (TLR, NOD, CLR, and RLR). The choice of proinflammatory cytokines incorporated into the maturation cocktails can lead to further optimization of cytokine-producing DC [62]. In line, NK cell-derived cytokines do have a decisive influence on DC-derived IL-12p70 production [17]. An increased IL-12p70 production can be achieved by simply adding higher concentrations of rhIFN-*γ* to a particular PRR-containing cocktail. This provides proof of principle that proinflammatory cytokines can be applied to fine-tune the maturation conditions.

Even though high IL-12-producing DC can be generated* in vitro* by manipulating the composition of the maturation cocktail, one crucial criterion of efficient induction of immune responses is the production of IL-12p70 (and other cytokines and chemokines)* in vivo* upon DC readministration into the patient. Usually cytokine measurements are performed on* in vitro* 24/48 h-matured DC. Most of the cytokines produced by moDC are released within the first 24 h [63]. In addition, we and others previously showed that in 6 h-matured DC the cytokine induction program is irreversibly primed [7, 64, 65]. Clinical trials employ diverse strategies to mature the DC. Several studies use 24 h/48 h-matured exhausted DC, which are not able anymore to produce IL-12p70 but regain this capacity after T cell encounter and the ligation of CD40. Others employ 6 h maturation protocols, generating semimature DC retaining the capacity to produce IL-12 even before the encounter with T cells* in vivo* [66]. The latter approach is favorable since DC should retain the capacity to produce NK cell-recruiting chemokines as well as NK cell-activating cytokines upon injection.

As diverse polymorphisms affect the IL-12p70 production of DC [67–70], another option is to engineer DC via the usage of mRNA, DNA, or recombinant viruses to constitutively produce IL-12. mRNA electroporation of DC has been shown to be efficient and a clinically safe transfection method has been described [71–73]. Another advantage of engineering DC is the specific selection of “desired” cytokines produced by DC without the production of anti-inflammatory cytokines, for example, IL-10 or TGF-*β*, or silencing undesired properties. Lipscomb et al. described an IL-12p70-independent mechanism for Th1 polarization when DC expressed ectopic TBX21 (T-bet) via adenoviral infection [74]. These findings were translated into engineering syngeneic TBX21 and IL-12p70 expressing DC. Injection of these DC into mice bearing subcutaneous tumors led to synergistic and robust antigen-specific type 1 immune responses including tumor rejection, crosspriming of Ag, and infiltration of CD8^+^ T cells [75]. Thus, engineering DC provides a multitude of intervention points [76] and displays a powerful approach to ensure long-lasting provision of cytokines, possibly in combination with other signals (enhancing stimulating or blocking negative modulators) in the tumor microenvironment (TME).

As shown previously [17], the cytokine and chemokine profile of moDC can also be enhanced by soluble factors derived from NK cells. In a similar approach, Berk et al. [77, 78] showed the possibility to use the supernatant of activated lymphocytes to induce maturation of DC including upregulation of phenotype markers, IL-12p70, and CXCL-10 production. These crosstalk features of DC with immune helper cells can be exploited to further boost potential of moDC. Although we showed the importance of high IL-12p70-producing moDC for the induction of Th1 and NK cell responses, also other cytokines were shown in several studies to contribute in an additive or synergic manner to improved helper responses (e.g., IL-15, IL- 18, and IFN-*α* [79, 80]).

Additionally, plasmacytoid DC (pDC) can also become activated by various PAMPs leading to the polarization of naive CD4^+^ T cells into Th1 cells [16]. pDC can produce IL-12p70; however, compared to moDC and myeloid DC, their IL-12p70 production level is very limited. Other cytokines have been shown to facilitate IFN-*γ* production, like IL-18, IFN-*α*, and IL-27 [79, 80]. Possibly, different DC subsets employ adjusted pathways to activate NK cells or Th1 cells. IFN-*α* secreted in high amounts by pDC was shown to induce TBX21 expression, although this pathway is less stable compared to IL-12 induction. It remains to be established whether the potency of the different subsets to polarize naive cells into Th1 cells is comparable or whether high IL-12-producing DC subsets favor this induction. Also for NK cell activation, a two-signal activation is much more effective [81, 82]. Likewise, IL-15 can potently enhance proliferation and survival of NK and T cells and enhance NK-DC crosstalk [83, 84]. Arguably, by choosing appropriate maturation stimuli, the DC cytokine profile can be fine-tuned, or DC can be engineered to produce the “optimal” cytokine combinations.

## 6. Importance of CD4^+^ T Cells: More Than Helpers? 

Numerous lines of evidence indicate the crucial role of CD4^+^ T cells in the generation of different aspects of adaptive immune responses. They are mainly important for the induction of potent CTL responses and for the generation of long-lived memory responses [85]. Furthermore, they also play an important role in modulating DC maturation by providing diverse cytokines. In mice, CD4^+^ T cells were shown to be required for improved tumor elimination by CD8^+^ T cells [86, 87]. CD4^+^ T cells enhanced the clonal expansion of CD8^+^ T cells in secondary lymphoid tissue after vaccination and tumor-specific CD4^+^ also facilitated recruitment, proliferation, and effector function of CD8^+^ into the TME by secretion of IFN-*γ* and IL-2. Therefore, it is widely assumed that immunotherapeutic approaches require the involvement of CD4^+^ T cells.* Ex vivo* maturation of DC should be directed to prime Th1 responses. With a newly developed assay [16], DC-mediated direction, potency, and kinetics of Th cell differentiation can be monitored. Results revealed that PGE_2_/TNF-*α* matured DC, which have been mostly used in clinical studies, induce a Th2-like response. Other differently matured DC promoted significant differences in their Th1 polarization capacity [8, 17].

Recently, the targeting of CD4^+^ T cells by vaccination with a polytope mRNA vaccine (encoding immunogenic mutant class II epitopes) has been shown to be very efficient in mice by meditating strong antitumor responses [88]. The vaccination led to reversal of suppression by the TME and to induction of CTL. Adding a human leukocyte antigen- (HLA-) class II targeting signal (DC-LAMP) to mRNA encoding tumor antigens will also activate Th1 and CTL responses [70, 73]. These findings highlight the importance of CD4^+^ T cells in immunotherapy and consist in a very promising approach to become part of the standard therapy in the clinic.

## 7. Importance of NK Cells

Whereas previous approaches to optimize DC vaccination were mainly based on maximizing intratumoral T cell responses, other players of the immune system may also be important in the process of tumor cell elimination. NK cells are able to exert direct cytotoxic effects on tumor cells or indirectly modulate the adaptive immunity by cytokine secretion and communication with other immune cells [40, 89–91]. Moreover, low cytolytic activity of NK cells has been associated with 40% increased cancer risk compared to individuals with NK cells having high cytolytic activity [92]. Likewise, levels of intratumoral NK cells and NK cell activity are positively correlated with clinical outcome [93–96]. In patients with cancer, NK cell functions are often impaired displaying reduced cytolytic and cytokine secreting capacities and reduced DC editing [97–100].

As NK cells and DC have a strong mutual interaction, it is plausible to devise strategies combining (actions of) these cells to overcome dysfunction and enhance antitumor responses. For instance, PAMP-stimulated NK cell-derived supernatant can be used in the preparation of DC [101, 102] to maximize their maturation. These findings are strengthened by our recent study describing that soluble factors derived from PAMP-activated NK cells did enhance the cytokine and chemokine profile of* ex vivo* matured moDC (Oth et al., manuscript under revision). We earlier proposed to optimize* ex vivo* maturation of DC in a way where they are able to optimally recruit and activate NK cells [17, 40]. The capacity of DC to efficiently interact with NK cells is influenced by multiple parameters like the differentiation and maturation of DC, as well as the choice and delivery of Ag. TLR agonists are potent and necessary components in the DC maturation process for optimal NK cell activation and recruitment [7, 102]. Also the cytokines used during the differentiation of monocytes (e.g., IFN-*α* or IL-15) can have an effect on the capacities of DC to recruit and activate NK cells [7, 103–107]. For instance, we have shown that PGE_2_ negatively regulates NK-DC crosstalk [57]. Of note, a study by Jensen et al. [62] investigating the effect of different combinations of recombinant human cytokines with PRR triggers revealed that PGE_2_ production by moDC is induced upon selected maturation stimuli. The DC maturation cocktails used in clinical trials often contain PGE_2_ to induce a migratory capacity of DC [108, 109] but do not avail as only 3–5% of injected DC reach the draining lymph nodes [110]. A combined NK-DC therapy may be more attractive. Antigenic material released by NK cell-killed tumor cells can be taken up and presented by DC. Moreover, NK cells can remove inappropriately matured or immature DC and mature DC may augment NK cell cytotoxicity.

The combination may also induce development of a tertiary lymphoid structure (TLS). The density of such lymphoid islets adjacent to tumors in combination with mature DC correlates with Th1/CTL tumor infiltrating phenotype and with positive clinical outcome [111, 112]. The administered DC will produce chemokines and, thereby, selectively recruit NK effector cells [8] as well as CTLs and Th1 cells [113–115]. We have previously hypothesized [40] that in the event DC recruit all these effector cells a TLS will be formed and replace the interactions normally taking place in lymph nodes. It remains to be established whether the TME suppresses the effector cell induction by DC* in vivo*.

NK-DC crosstalk, however, exerts not only immunostimulatory effects. In this line, a recent study of Sarhan et al. [116] showed that NK-DC crosstalk is inhibited in the presence of IL-2 affecting NK cell-derived IFN-*γ* production, cytolytic activity, and proliferation. This effect is indirectly mediated by the negative effect of IL-2 on DC-derived IL-12 and lymphotoxin alpha secretion due to STAT3 phosphorylation. Because NK helper cells will mostly interact with DC in the lymph nodes surrounded by naive T cells and Th1 cells and thus IL-2, this is an important aspect to consider for vaccination strategies.

## 8. Importance of CD8^+^ T Cells

CD8^+^ CTL cells are very important effector cells in clearing tumors. It is thus no surprise that the first DC vaccines that were developed focused on MHC class I peptide loaded DC to activate CD8^+^ T cells (e.g., [117, 118]), this is a sum-up of three parameters, CTL in* in vitro* models, CTL in* in vivo* models (mice), and CTL in biopsies from cancer patients. Several reasons can be indicated for this, but the fact that helper responses are needed for CTLs to stimulate their maturation and improve their killing capacity [119] is beyond doubt. The required help is traditionally provided by CD4^+^ T cells [120] but also NK cells can provide help [82, 121]. Solutions for the design of DC vaccines lie in the addition of class II targeting sequences as the invariant chain [122] or including DC-LAMP in the mRNA to be transfected [123], resulting in stronger help for CD8^+^ T cells.

## 9. Importance of CD4^+^ Regulatory T Cells

Chemokines released by tumor cells and immune cells present in microenvironment attract also lymphocytes (tumor infiltrating lymphocytes (TIL)). Low numbers of CD8^+^ TIL and high number of Treg TIL are associated with poor prognosis [124]. The presence of abundant numbers of Treg in the tumor, tumor-draining lymph nodes, and peripheral blood is one of the interfering components hampering DC-induced activation and expansion of type-1 immune responses [125]. Treg can efficiently suppress innate and adaptive arms of antitumor immune responses on multiple levels. Hence, the depletion or functional modulation of these cells is a possible way to restore the immunosuppression.

Furthermore, it is important to check whether DC vaccination does not induce Tregs. It is likely that, by proper stimulation of DC, polarisation of naive T cells into Tregs will not occur. In the polarization assays with FMKp/IFN-*γ* matured DC and naive CD4^+^ T cells, we did not detect them (unpublished data).

## 10. Combination Therapies: Necessity of Multileveled Therapies

Even though in a majority of patients an increased immune response was observed after DC vaccine administration, this effect was not yet reflected in the overall outcome. Many clinical trials applying optimized DC-based vaccines are currently ongoing. However, the direct effect of the TME on DC and the indirect effect on the DC-activated immune effector cells remain a major hurdle in therapeutic DC vaccine anticancer strategies and cancer immunotherapy in general. There is growing evidence that the host's immune systems play a crucial role in tumor progression [112, 126–128] and that the clinical outcome of treatment is dependent on the patients' TME acting as rheostat on induced immune responses. In this line, patients at the same stage of disease do display different clinical outcomes after intervention [129]. Different approaches are being explored to turn the immunosuppressive environment into an immunosupportive milieu, but they should limit a chronic inflammatory state and thus avoid production of high amounts of TNF-*α*, IL-1, IL-6, and IL-8.

Another escape phenomenon that should be considered is immune editing. On each time an anticancer treatment induces a potent antitumor response by inducing diverse immune effector cells the pressure on the tumor cells to adapt and to survive is increased. Thus, each treatment will induce partial resistance of the tumor due to its heterogeneity and lead to selective outgrowth of surviving cells (less immunogenic cells). As such, the tumor adapts its phenotype over time [130–134].

The key to success of immunotherapy will most probably be to circumvent the inhibition and escape mechanisms of the tumor. Whereas various single targeted approaches have shown partial success in tumor remission or increase in overall survival, the solution may be not only a multileveled treatment approach combining nonspecific (like adjuvants, cytokines, and checkpoint inhibitors) and specific treatment regimens (antibodies and vaccination) but also including conventional anticancer therapies (radiotherapy and chemotherapy). In particular checkpoint inhibitors have gained great attention. Monoclonal antibodies against inhibitory molecules expressed on T cells like CTLA-4 and PD-1 block the brake of the immune system, resulting in longer lasting immune responses. Although very encouraging clinical results have been obtained as recently reviewed by Mahoney et al. [135], the treatment is still accompanied by toxicity issues [136] that remain to be solved.

Depletion or functional modulation of Treg is a possible way to restore the immunosuppression. Treg are characterized as CD4^+^CD25^+^CCR4^+^GITR^+^. The depletion can be achieved with mAb against CD25. However, this also affects other (effector) T cell populations which upregulate CD25 as a consequence of their activation [137, 138]. The chemokine receptor CCR4 is highly expressed on effector Treg cells and displays low expression on naive Treg and non-Treg cells [139], making it an interesting target to deplete Treg by using anti-CCR4 mAb. Application of agonist mAb against GITR and OX40, respectively, led to attenuation of suppressive function of Treg and increased effector antitumor T cell functions in several studies [140–144]. The blocking of Treg by stimulating OX40 or GITR to reverse immunosuppressive milieu in the tumor may be a more safe approach than depleting Treg.

One of the biggest challenges in immunotherapy is the lack of biomarkers predicting when to apply which therapy. If tumor biopsies are available, in addition to histopathology, immunophenotyping should also be performed because of the involvement of the immune host defense in tumor progression. This approach was defined as “immunoscore” [145] and consists in detecting TIL in the center and invasive margin of the tumor (number of CD3/CD8 or CD8/CD45RO). As TIL are heterogeneous between tumors and patients, this analysis of immune contexture will help give a better prognosis and make better clinical decisions [146]. Patients with a low immunoscore, meaning low infiltration of CD8^+^ T cells in the tumor, would be good targets for adjuvant therapy to increase immunogenicity of the tumor.

Another key factor is the tumor burden at the start of the intervention. Low tumor burden seems to be more sensitive to immunotherapeutic approaches. Likewise, an initial tumor treatment approach with conventional therapies may be necessary to remove the majority of the tumor burden. A possible option for applying DC-based vaccines is considering a basic treatment approach around a DC-based vaccine, which has to be adjusted and complemented with different combination strategies depending on both histopathological features as well as the characterization of the tumor microenvironment of the patient (if possible). The rationale for combination therapies is also illustrated in ongoing clinical trials applying DC vaccine strategies and as such DC vaccine in combination with anti-CTLA-4 is currently evaluated in clinical trials [147]. Targeting PD-1 or PD-L1 may also be an interesting combination [148, 149]. In mice, complete eradication of tumors by CD8^+^ CTLs has been reported after DC vaccine was combined with checkpoint inhibitors [150].

A general prerequisite for the application of checkpoint inhibitors is the immunogenicity of tumors. Presumably this immunogenicity can and should be enhanced locally, for example, by the (intratumoral) administration of certain PRR triggers inducing direct toxicity on tumor cells and generating an immunosupportive environment. Also chemotherapy agents as well as radiotherapy will continue to have a crucial role in the preconditioning of the tumor. The subsequent administration of DC vaccine would enhance antitumor specific responses. Once initiated, blocking, for example, PD-L1 could retain antitumor specific cells in an active state. However, timing will be a crucial factor in any multileveled approach.

## 11. Conclusions

The interaction of DC with both Th1 and NK cells revealed that high IL-12p70 secreting DC have the capacity to activate both helper responses, resulting in larger and stronger killing capacity by CD8^+^ CTL. Additionally, NK cells act as amplifiers to enhance cytokine and chemokine production by DC needed for T and NK cells attraction and activation. Furthermore, one of the mechanisms by which tumor environment inhibits immune responses is the blocking of NK-DC crosstalk. Successful combinations of PAMP triggers to mature DC showing enhanced capacities to interact with NK cells and to induce Th1 polarization* in vitro* have been identified. There are important criteria that should be taken into account when selecting PAMPs as adjuvants for vaccination. Multiple factors explain the so far overall limited clinical outcome of immunotherapy and specifically of DC-based vaccination. Combinations of immunotherapy, including checkpoint inhibitors, with chemotherapy and/or radiotherapy will yield better results, overcoming the suppressive TME by attacking multiple pathways to initiate and elongate desired antitumor immune responses.
